# Biotechnological Potential of Rhizospheric *Bacillus* Strains from Lonquimay, Chile, as Producers of Antimicrobial Biosurfactants

**DOI:** 10.3390/ijms27125401

**Published:** 2026-06-15

**Authors:** Claudio Lamilla, Olga Rubilar, Ignacio San Martin, David Troncoso, Sebastián Rojas, Daniel Martínez-Cisterna, Diana L. Cárdenas-Chávez, María Cristina Diez, Andrés Quiroz

**Affiliations:** 1 Scientific and Technological Bioresource Nucleus (BIOREN), Universidad de La Frontera, Av. Francisco Salazar 01145, Casilla 54-D, Temuco 4811230, Chile; 2 Centro de Investigación Biotecnológica Aplicada al Medio Ambiente (CIBAMA), Universidad de La Frontera, Av. Francisco Salazar 01145, Casilla 54-D, Temuco 4811230, Chile; olga.rubilar@ufrontera.cl (O.R.); daniel.martinez@ufrontera.cl (D.M.-C.); cristina.diez@ufrontera.cl (M.C.D.); 3 Departamento de Ingeniería Química, Facultad de Ingeniería y Ciencias, Universidad de La Frontera, Av. Francisco Salazar 01145, Temuco 4780000, Chile; 4 Universidad de La Frontera, Av. Francisco Salazar 01145, Casilla 54-D, Temuco 4811230, Chile; i.sanmartin09@ufromail.cl; 5 Programa de Doctorado en Ciencias de Recursos Naturales, Universidad de La Frontera, Av. Francisco Salazar 01145, Casilla 54-D, Temuco 4811230, Chile; d.troncoso03@ufromail.cl; 6 Programa de Magíster en Ciencias de la Ingeniería Mención Biotecnología, Universidad de La Frontera, Av. Francisco Salazar 01145, Casilla 54-D, Temuco 4811230, Chile; sebastianmarcelo.rojas@ufrontera.cl; 7 Tecnologico de Monterrey, School of Engineering and Sciences, Vía Atlixcáyotl 5718, Puebla 72453, Mexico; diana.cardenas@tec.mx; 8 Laboratorio de Química Ecológica, Departamento de Ciencias Químicas y Recursos Naturales, Universidad de La Frontera, Av. Francisco Salazar 01145, Casilla 54-D, Temuco 4811230, Chile

**Keywords:** antibacterial activity, antifungal activity, bacillus, surfactin

## Abstract

Biosurfactants are surface-active microbial molecules with increasing industrial relevance as sustainable alternatives to synthetic surfactants. Among them, lipopeptides produced by *Bacillus* species, particularly surfactin, exhibit strong interfacial activity and biological functionality. In this study, rhizospheric soils from the La Araucanía region, Chile, were explored as a source of biosurfactant-producing bacteria. Eighteen strains were isolated, and two high-performing strains, Solo 1 and Solo 4, were identified as *Bacillus amyloliquefaciens* and *Bacillus subtilis*, respectively. Both strains harbored the *srfAA* gene and produced surfactin isoforms confirmed by MALDI-TOF MS. Kinetic analysis revealed distinct production profiles, with Solo 1 reaching a maximum of 90 mg L^−1^ at 24 h, whereas Solo 4 showed continuous production up to 224.4 mg L^−1^ at 72 h. Both biosurfactants exhibited high emulsification capacity (>80%) and stability across wide ranges of temperature, pH, and salinity. Importantly, cell-free supernatants from both strains showed antibacterial and antibiofilm activity against *Staphylococcus aureus*, with Solo 4 reaching 81% biofilm inhibition. In addition, surfactin-enriched extracts inhibited the pathogenic bacterium *Pseudomonas syringae* and the filamentous fungus *Fusarium oxysporum*, with Solo 4 consistently showing stronger antimicrobial performance. Overall, these findings identify Solo 4 as a promising native *Bacillus* strain for future development of biosurfactant-based systems aimed at antimicrobial control, biofilm management, agricultural pathogen suppression, surface sanitation, and environmentally compatible biotechnological processes.

## 1. Introduction

Biosurfactants are a diverse group of surface-active compounds produced by microorganisms, including bacteria, fungi, and yeasts [1]. These natural molecules possess unique properties that enable them to lower the surface tension between liquids and immiscible phases, such as water and oil [2]. Unlike synthetic surfactants derived from petrochemical sources, biosurfactants are biodegradable, environmentally friendly, and exhibit high compatibility under variable temperature, pH, and salinity conditions [3]. Growing environmental concerns and regulatory pressure have increased interest in biosurfactants as sustainable alternatives for diverse industrial sectors [3,4,5].

The functional versatility of biosurfactants arises from their amphiphilic structure, which enables mechanisms such as emulsification, solubilization, and dispersion, leading to improved hydrocarbon desorption, enhanced oil displacement, and more efficient nutrient transfer in environmental systems [6,7,8,9]. Due to these properties, biosurfactants have been investigated in several application domains, including environmental remediation [6], enhanced oil recovery (EOR) [7], cosmetics, food processing, agrochemicals, and pharmaceuticals [8]. In industrial settings, biosurfactants play a role in foam formation, detergency, cleaning-in-place (CIP) operations, and formulation stability [9]. The mode of action of biosurfactants includes emulsification, solubilization, and dispersion, which can lead to improved oil recovery from reservoirs, the degradation of hydrophobic pollutants in the environment, and the enhancement of nutrient uptake in plants [10]. In addition, the ability of biosurfactants to disrupt biofilms can aid in combating bacterial infections and improving the efficacy of antimicrobial agents [8,11,12]. Overall, the sustainable and eco-friendly nature of biosurfactants fosters their integration into emerging green technologies, such as bioremediation of contaminated soils and waters, enhanced oil recovery operations, and sustainable cleaning formulations [13,14,15,16].

Among biosurfactant-producing microorganisms, species of the genus *Bacillus* have gained considerable attention due to their ability to produce a wide range of biosurfactants, including lipopeptides, glycolipids, and lipoproteins [17,18]. Lipopeptides such as surfactin, iturin, and fengycin are considered among the most promising compounds due to their strong interfacial activity, antimicrobial properties, and potential for environmental remediation [19]. Surfactin has been particularly studied for its ability to enhance biodegradation of petroleum-derived pollutants by increasing their bioavailability to microbial degraders, and its surface-active properties have been exploited for EOR applications by altering oil–water interfacial tension and enhancing oil displacement from reservoirs [20,21]. Moreover, surfactin exhibits antimicrobial activity, supporting applications in biofouling control across industrial and biomedical domains. Its emulsifying and foaming properties have also encouraged adoption in cosmetics and personal care formulations, while its safety profile has enabled use in food processing as an emulsifier and stabilizer [22,23]. From a biotechnological perspective, surfactin has been used in cell culture processes, protein purification, and nanoparticle synthesis [24,25].

*Bacillus*-derived lipopeptides are also relevant due to their antimicrobial activity against clinically and agriculturally important microorganisms. *Staphylococcus aureus* is a Gram-positive bacterium widely used as a model pathogen in antimicrobial and antibiofilm studies because of its ability to adhere to surfaces and form persistent biofilms, which are associated with increased tolerance to antimicrobial treatments. Recent evidence has shown that surfactin-containing biosurfactants from *Bacillus* strains can inhibit *S. aureus* growth, biofilm formation, and virulence-associated traits, supporting their potential as alternative antimicrobial agents [26]. In agricultural contexts, *Pseudomonas syringae* represents a relevant Gram-negative phytopathogenic bacterium, while *Fusarium oxysporum* is among the most important filamentous phytopathogenic fungi associated with vascular wilt, root diseases, and crop losses. Recent studies have emphasized that cyclic lipopeptides from *Bacillus*, including surfactin, iturin, and fengycin families, participate in rhizosphere microbial interactions, plant protection, and antagonism against phytopathogens [27,28,29,30]. Therefore, evaluating biosurfactant and surfactin activity against Gram-positive, Gram-negative, and fungal targets provides a broader perspective on their potential use in microbial control strategies for biomedical, industrial, and agricultural contexts.

Despite the increasing interest in biosurfactants, commercial implementation remains limited due to production costs, substrate requirements, and variable yields among biosurfactant-producing microorganisms [31]. In this context, the identification of native microbial strains capable of producing biosurfactants with industrially relevant physicochemical properties and adequate production performance represents a key biotechnological step toward enabling economically viable biosurfactant production systems, particularly rhizospheric niches [32]. Soil environments, particularly rhizospheric niches, constitute promising microbial reservoirs for bioprospecting due to their high microbial diversity and metabolic versatility [33].

In this study, rhizospheric soils from La Araucanía region of Chile were explored as a biological source for biosurfactant-producing bacteria. Eighteen bacterial strains were isolated and screened for biosurfactant activity, and two high-performing strains were selected for molecular identification, biosurfactant characterization, stability assessment, and antimicrobial evaluation against *Staphylococcus aureus*, *Pseudomonas syringae*, and *Fusarium oxysporum*. The biotechnological properties demonstrated by these native *Bacillus* strains support their potential use in industrial, environmental, biomedical, and agricultural processes.

## 2. Results

### 2.1. Isolation and Preliminary Screening of Biosurfactant-Producing Strains

A total of 18 bacterial strains were isolated from rhizospheric soil based on colony morphology. The morphological characteristics of the strains are summarized in Table 1. Hemolytic activity was evaluated on sheep blood agar plates as a preliminary screening criterion for biosurfactant production. Six strains exhibited positive hemolysis, characterized by clear zones around the colonies, and were selected for subsequent biosurfactant production assays.

### 2.2. Biosurfactant Production

**Figure 1:**
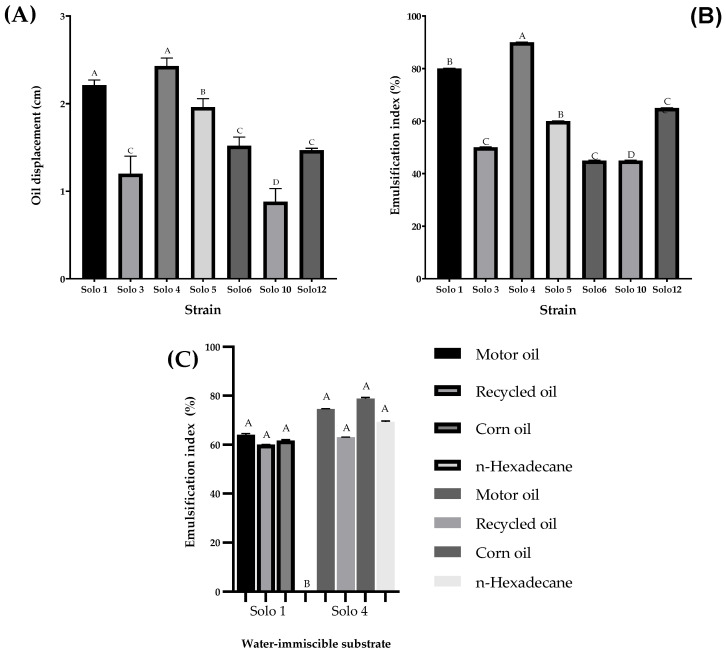
Preliminary evaluation of biosurfactant production and emulsifying activity of selected bacterial strains using cell-free supernatants: (**A**) Oil displacement activity expressed as halo diameter (cm). (**B**) Emulsification index (E24, %) of selected biosurfactant-producing strains. (**C**) Emulsification index (E24, %) of cell-free supernatants evaluated against different water-immiscible substrates. Data are shown as mean ± SD. Different letters indicate significant differences among treatments within each panel (*p* < 0.05).

Biosurfactant production was evaluated by oil displacement and emulsification assays (Figure 1). The supernatants of strains Solo 1 and Solo 4 showed the highest oil displacement values, exceeding 2 cm (Figure 1A). Similarly, both strains exhibited emulsification indices above 80%, outperforming the other strains (Figure 1B). Based on these results, strains Solo 1 and Solo 4 were selected for subsequent characterization and application assays.

To further evaluate the emulsifying capacity of the selected biosurfactant-producing strains, cell-free supernatants from Solo 1 and Solo 4 were tested against different water-immiscible substrates, including motor oil, recycled oil, corn oil, and n-hexadecane (Figure 1C). Both strains showed emulsifying activity against motor oil, recycled oil, and corn oil, with emulsification index (E24) values above 60%. However, their response to n-hexadecane differed markedly: no emulsification was detected for Solo 1, whereas Solo 4 maintained high emulsifying activity. These results indicate that the emulsifying performance of the biosurfactants depends on both the producing strain and the hydrophobic substrate used.

### 2.3. Molecular Identification of Selected Strains

**Figure 2:**
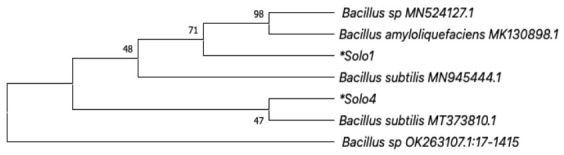
Phylogenetic relationships of the selected biosurfactant-producing strains based on 16S rRNA gene sequences.

The two selected strains were identified by 16S rRNA gene sequencing. Phylogenetic analysis showed that strain Solo 1 clustered within the *Bacillus amyloliquefaciens* clade, whereas strain Solo 4 grouped within the *Bacillus subtilis* clade (Figure 2). The sequences were deposited in GenBank under accession numbers OR424730 (Solo 1) and OR424731 (Solo 4).

### 2.4. Detection of the Surfactin Biosynthesis Gene srfAA

**Figure 3:**
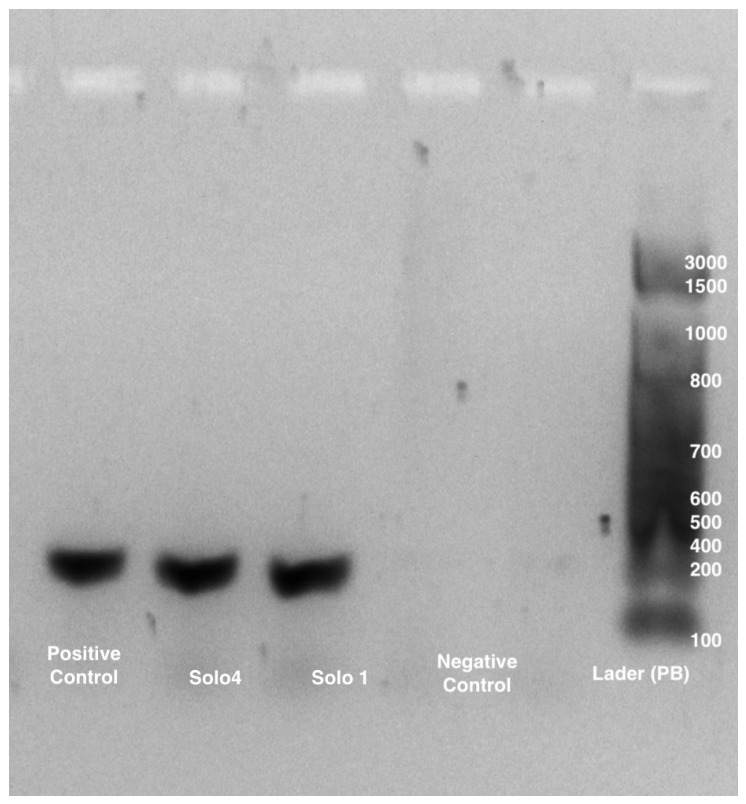
Agarose gel electrophoresis of PCR-amplified fragments of the surfactin biosynthesis gene (*srfAA*).

PCR amplification revealed the presence of the *srfAA* gene in both Solo 1 and Solo 4, producing a fragment of approximately 200 bp, consistent with the expected amplicon size for surfactin synthetase genes (Figure 3).

### 2.5. Kinetics of Biosurfactant Production and Surfactin Concentration

**Figure 4:**
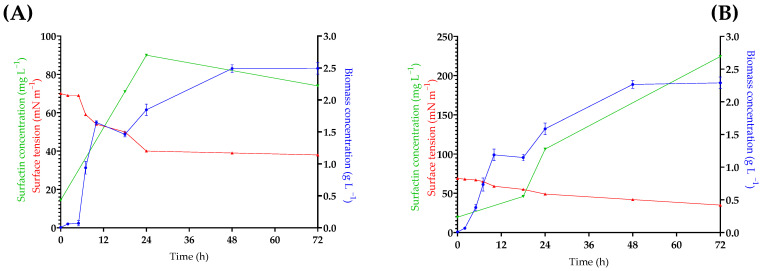
Kinetics of biomass formation, surfactin production, and surface tension reduction by strains Solo 1 and Solo 4 in LB medium: (**A**) Strain Solo 1. (**B**) Strain Solo 4. Biomass concentration (g L^−1^, blue), surfactin concentration determined by HPLC (mg L^−1^, green), and surface tension (mN m^−1^, red). Data are given as the mean ± SD.

The growth kinetics, surfactin production, and surface tension reduction of strains Solo 1 and Solo 4 are shown in Figure 4. Both strains entered the exponential phase after approximately 8–10 h of incubation, followed by a progressive increase in biomass concentration. For strain Solo 1, biomass reached 2.5 g L^−1^ at 48 h and remained relatively stable thereafter (Figure 4A). In strain Solo 4, biomass increased continuously up to 72 h, reaching 2.7 g L^−1^ (Figure 4B).

Surface tension decreased during cultivation in both strains, indicating biosurfactant production. In Solo 1, surface tension declined from approximately 70 to 40 mN m^−1^ by 24 h and remained relatively stable thereafter. In Solo 4, surface tension decreased more gradually, from 69 to 35 mN m^−1^ at 72 h.

High-performance liquid chromatography (HPLC) analysis revealed that surfactin production differed markedly between the two strains. Solo 1 reached a maximum surfactin concentration of 90 mg L^−1^ at 24 h, after which the concentration gradually decreased to 74 mg L^−1^ at 72 h (Figure 4A). In contrast, Solo 4 showed a continuous increase in surfactin concentration throughout cultivation, reaching a maximum of 224.4 mg L^−1^ at 72 h (Figure 4B). These results indicate that Solo 4 was the more efficient surfactin producer under the evaluated conditions.

### 2.6. Physicochemical Stability of Biosurfactants

**Figure 5:**
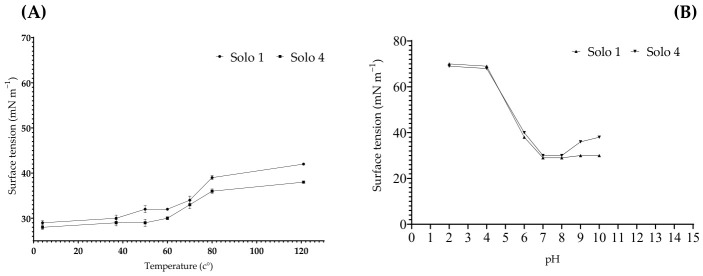
Physicochemical stability of biosurfactants produced by strains Solo 1 and Solo 4 under temperature and pH conditions. (**A**) Effect of temperature (4–122 °C) on surface tension. (**B**) Effect of pH (2–10) on surface tension. Data are given as the mean ± SD.

**Figure 6:**
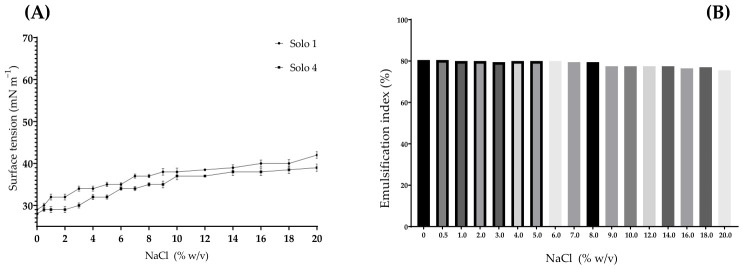
Effect of salinity on the surface activity and emulsifying capacity of biosurfactants produced by strains Solo 1 and Solo 4. (**A**) Effect of NaCl concentration (1–20%, *w*/*v*) on surface tension. (**B**) Emulsification index (E24, %) under different NaCl concentrations (1–20%, *w*/*v*). Data are given as the mean ± SD.

The stability of the biosurfactants produced by strains Solo 1 and Solo 4 was assessed under varying temperature, pH, and salinity conditions. As shown in Figure 5A, both biosurfactants remained active between 4 and 70 °C. Surface activity was maintained between pH 6 and 10 (Figure 5B), with Solo 1 exhibiting greater tolerance at pH 9–10 compared to Solo 4. Salinities ranging from 1% to 5% (*w*/*v*) NaCl also supported biosurfactant activity (Figure 6A). However, NaCl concentrations of 10% (*w*/*v*) or higher significantly reduced surface activity, with ST values dropping below 42 mN m^−1^ in both strains. Notably, Solo 4 maintained relatively consistent activity across the evaluated salinity range. In agreement with these results, the emulsification index remained relatively stable across the tested NaCl concentrations for both strains (Figure 6B), indicating that salinity had only a limited effect on emulsion-forming capacity under the evaluated conditions.

### 2.7. Chemical Characterization by MALDI-TOF MS

**Figure 7:**
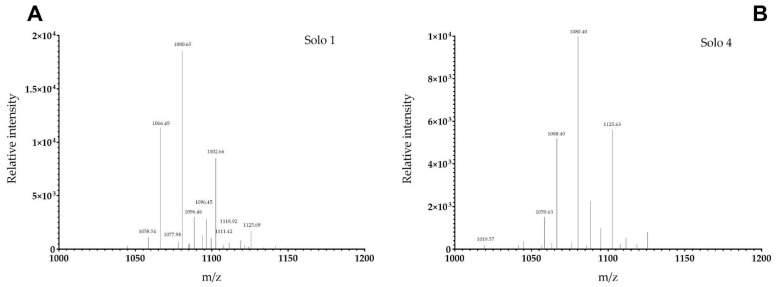
MALDI-TOF/TOF mass spectra of surfactin-enriched lipopeptide fractions from Strains Solo 1 and Solo 4: (**A**) Mass-to-charge (*m*/*z*) spectrum of lipopeptides obtained from strain Solo 1. (**B**) Mass-to-charge (*m*/*z*) spectrum of lipopeptides obtained from strain Solo 4.

The mass-to-charge ratios (*m*/*z*) of the main lipopeptide peaks are shown in Figure 7. In Figure 7A, the spectrum of strain Solo 1 reveals the most intense peak at 1102.66 *m*/*z*, suggesting that this surfactin isoform is the most abundant in the Solo 1 sample. Additional peaks at 1080.65, 1066.49, and 1058.54 *m*/*z* indicate the presence of further isoforms. For strain Solo 4, the most intense peak is observed at 1125.63 *m*/*z*, indicating a slightly different dominant isoform compared to Solo 1. A secondary intense peak appears at 1080.40 *m*/*z*, aligning with one of the Solo 1 peaks. Additional peaks at 1059.63 *m*/*z* and 1019.57 *m*/*z* reflect a range of surfactin isoforms similar to those in Solo 1, though with some differences in abundance and distribution.

### 2.8. Antibacterial and Antibiofilm Activity of Cell-Free Supernatants Against Staphylococcus aureus

#### 2.8.1. Growth Inhibition Assay

**Figure 8:**
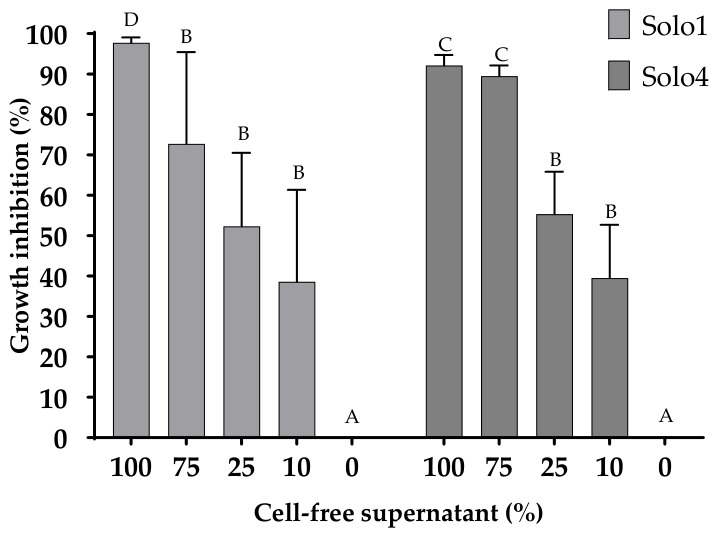
Antibacterial activity of cell-free supernatants from strains Solo 1 and Solo 4 against *Staphylococcus aureus*. Different letters above the bars indicate significant differences among treatments.

The antimicrobial activity of cell-free supernatants from strains Solo 1 and Solo 4 against *S. aureus* is shown in Figure 8. A clear concentration-dependent inhibitory effect was observed for both strains, with higher supernatant concentrations producing greater inhibition.

At 100% supernatant concentration, growth inhibition reached 97.7% for Solo 1 and 92.1% for Solo 4, indicating strong antibacterial activity. At 75% concentration, Solo 4 maintained a higher inhibitory effect (89.5%) compared to Solo 1 (72.7%). At lower concentrations, inhibition progressively decreased, reaching 52.3% and 38.6% for Solo 1 at 25% and 10% concentrations, respectively, and 55.3% and 39.5% for Solo 4 under the same conditions.

Overall, both supernatants exhibited strong antibacterial activity against *S. aureus*, with Solo 4 retaining greater inhibitory capacity after dilution, particularly at intermediate concentrations. These findings suggest that the extracellular fraction of Solo 4 contains bioactive metabolites with greater antibacterial persistence under reduced concentration conditions.

#### 2.8.2. Antibacterial Activity by Agar Diffusion Assay

**Figure 9:**
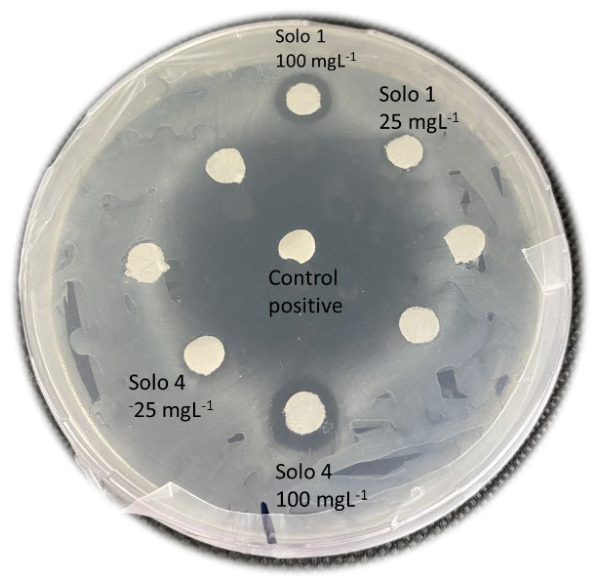
Representative agar diffusion assay illustrating the antimicrobial activity of cell-free supernatant samples from strains Solo 1 and Solo 4 against *Staphylococcus aureus*.

The agar diffusion assay confirmed the antibacterial activity of cell-free supernatant samples from both strains against *S. aureus* (Figure 9). The size of the inhibition halos increased with biosurfactant concentration, indicating a concentration-dependent response.

At 25 mg L^−1^, Solo 1 produced an inhibition zone of 12 mm, while Solo 4 reached 18 mm. At 100 mg L^−1^, inhibition zones increased to 20 mm for Solo 1 and 30 mm for Solo 4. The positive control (florfenicol, 8000 mg L^−1^) produced a halo of 40 mm.

Relative antimicrobial activity, expressed as a percentage of the positive control, reached 30% and 50% for Solo 1 at 25 and 100 mg L^−1^, respectively, whereas that of Solo 4 reached 45% and 75% under the same conditions. These results confirm that Solo 4 exhibited stronger antibacterial activity than Solo 1 (Table 2).

#### 2.8.3. Antibiofilm Activity on Silicone Tubes

**Figure 10:**
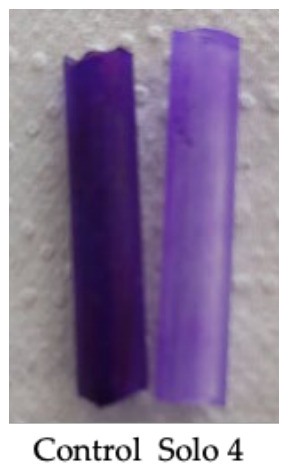
Antibiofilm activity of the cell-free supernatant from strain Solo 4 against *Staphylococcus aureus* on silicone tubes. Biofilm formation was visualized by crystal violet staining.

The antibiofilm assay revealed a strong inhibitory effect of the cell-free supernatants from strain Solo 4 on *S. aureus* biofilm formation (Figure 10). The untreated control exhibited intense crystal violet staining, corresponding to high biofilm formation, with replicate scores of ++++, ++++, and +++, resulting in a mean value of 3.7 (Table 3).

In contrast, treatment with the cell-free supernatants from strain Solo 4 markedly reduced biofilm formation, with replicate scores of +, +, and -, corresponding to a mean value of 0.7. Based on these values, the estimated biofilm inhibition reached approximately 81.1%.

These results indicate that the cell-free supernatant from Solo 4 was effective not only in inhibiting planktonic bacterial growth but also in reducing surface-associated biofilm formation.

### 2.9. Antimicrobial Activity of Surfactin-Enriched Extracts

#### 2.9.1. Antibacterial Activity Against *Pseudomonas syringae*

**Figure 11:**
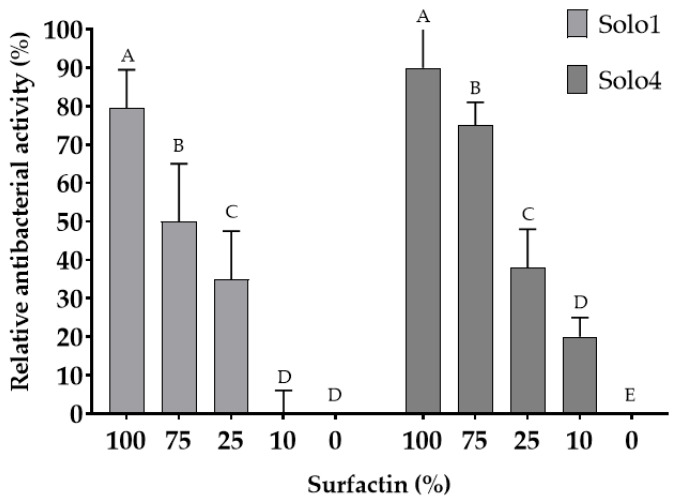
Antibacterial activity of surfactin-enriched extracts from strains Solo 1 and Solo 4 against *Pseudomonas syringae*. Activity was expressed as relative activity compared with the florfenicol control. Different letters above the bars indicate significant differences among treatments.

The antibacterial activity of surfactin-enriched extracts obtained from strains Solo 1 and Solo 4 against *Pseudomonas syringae* was evaluated by agar diffusion assay (Figure 11). A concentration-dependent inhibitory effect was observed for both surfactin-enriched samples, with higher concentrations producing stronger antibacterial activity.

At 100% extract concentration, inhibition reached 79.5% for Solo 1 and 90% for Solo 4, indicating strong antibacterial activity against *P. syringae*. At 75% concentration, Solo 4 maintained higher activity (75%) than Solo 1 (50%). At lower concentrations, antibacterial activity decreased progressively, reaching 35% for Solo 1 and 38% for Solo 4 at 25% concentration. At the lowest concentration (10%), Solo 1 showed no detectable inhibitory activity, whereas Solo 4 still retained 20% relative inhibition (Table 4).

Overall, surfactin-enriched extracts from both strains showed marked antibacterial activity against *P. syringae*, with Solo 4 exhibiting greater inhibitory persistence under dilution conditions. These results reinforce the functional superiority of the Solo 4 extract and suggest a broader antimicrobial potential against phytopathogenic bacteria.

#### 2.9.2. Antifungal Activity Against *Fusarium oxysporum*

**Figure 12:**
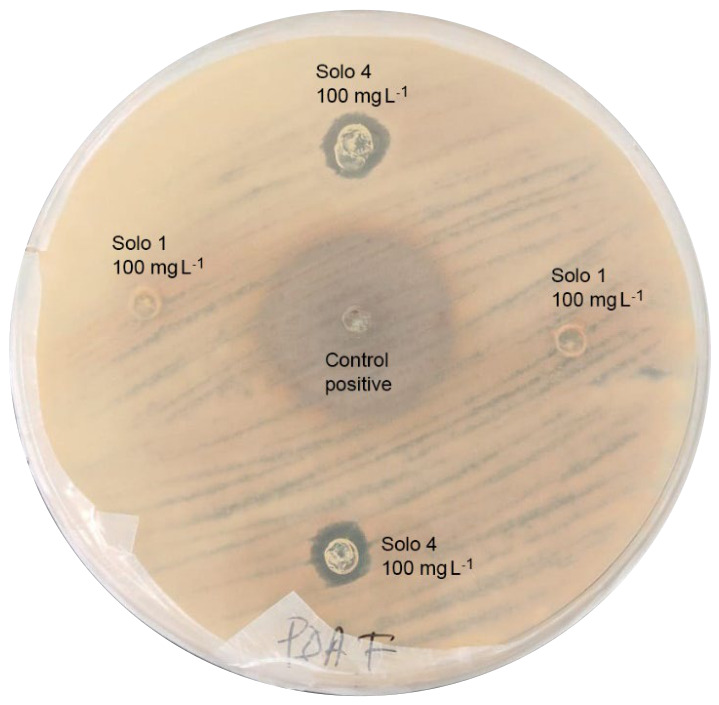
Representative agar diffusion assay illustrating the antifungal activity of surfactin-enriched extracts from strains Solo 1 and Solo 4 against *Fusarium oxysporum.* Copper oxychloride was used as a fungicide reference treatment and did not produce a visible inhibition zone under the evaluated conditions.

The antifungal assay demonstrated that surfactin-enriched extracts from strains Solo 1 and Solo 4 inhibited the growth of *Fusarium oxysporum*, as evidenced by the formation of inhibition zones surrounding the treatment wells (Figure 12). In contrast, the copper oxychloride reference treatment did not produce visible inhibition, suggesting reduced sensitivity or resistance of the fungal isolate under the experimental conditions.

At 100 mg L^−1^, Solo 1 produced an inhibition zone of 5.2 mm, while Solo 4 produced a larger inhibition zone of 15.29 mm, indicating stronger antifungal activity. The greater inhibition observed for Solo 4 is consistent with its higher surfactin production and stronger antimicrobial performance observed against bacterial pathogens.

These findings indicate that surfactin-enriched extracts obtained from the selected *Bacillus* strains possess antifungal activity against *F. oxysporum* and may represent a promising alternative for biological control of phytopathogenic fungi (Table 5).

## 3. Discussion

This study demonstrates that rhizospheric soils from the La Araucanía region represent a valuable reservoir of biosurfactant-producing bacteria with relevant biotechnological traits. From a total of 18 strains, six exhibited hemolytic activity, and two strains, Solo 1 and Solo 4, were selected based on their superior oil displacement and emulsification performance. This stepwise selection strategy is consistent with classical screening workflows used to identify promising biosurfactant-producing bacteria from environmental samples, where hemolytic activity is commonly used as an initial indicator and is subsequently complemented by functional surfactant assays such as oil displacement and emulsification index determination. In this context, the high performance of Solo 1 and Solo 4 in both assays supports their selection as candidates for further molecular, physicochemical, and biological characterization.

The identification of Solo 1 as *Bacillus amyloliquefaciens* and Solo 4 as *Bacillus subtilis* is particularly relevant from a biotechnological perspective. Species belonging to the genus *Bacillus* are among the most frequently reported biosurfactant producers, especially of lipopeptides such as surfactin, iturin, and fengycin, which are characterized by broad functional versatility, environmental robustness, and biological activity [34]. These species have also been associated with plant-growth promotion, antagonism against phytopathogens, and the production of metabolites of agricultural and industrial interest [35]. Therefore, the recovery of these taxa from rhizospheric soil is not unexpected, since the rhizosphere is a metabolically dynamic niche where microbial competition, root exudates, and physicochemical heterogeneity may favor the selection of strains able to produce extracellular compounds involved in colonization, nutrient acquisition, and ecological fitness [36,37].

The strong emulsification and oil displacement responses observed in Solo 1 and Solo 4 indicate efficient extracellular production of surface-active metabolites. This is consistent with previous reports describing *Bacillus*-derived biosurfactants as potent emulsifiers due to their amphiphilic structure and their ability to interact with hydrophobic substrates [38]. From an applied perspective, this behavior is highly relevant because the ability to reduce interfacial tension and stabilize emulsions underlies many of the technological uses proposed for microbial biosurfactants, including hydrocarbon mobilization, detergent formulations, cleaning systems, enhanced oil recovery, and treatment of hydrophobic contaminants [9,39]. In addition, the recovery of extracellular biosurfactant activity in culture supernatants is advantageous for downstream processing, since extracellular products are generally easier to recover than intracellular metabolites [40].

The presence of the *srfAA* gene in both strains provides molecular support for the biosynthetic potential of Solo 1 and Solo 4 to produce surfactin-like lipopeptides [41]. Since *srfAA* encodes part of the non-ribosomal peptide synthetase complex involved in surfactin biosynthesis, its detection strengthens the interpretation that the observed surface-active properties are associated with surfactin production [42]. However, the presence of the gene alone does not fully explain differences in performance between the two strains, because transcriptional regulation, precursor availability, post-synthetic modification, and strain-specific metabolic allocation may all influence final biosurfactant yield and activity [43]. This is particularly relevant in the present study, where both strains carried the same biosynthetic marker yet showed different kinetic and functional profiles.

The MALDI-TOF/TOF analysis further supports the production of surfactin-related lipopeptides and reveals compositional differences between strains. Solo 1 showed a dominant peak at 1102.66 *m*/*z*, whereas Solo 4 displayed its most intense peak at 1125.63 *m*/*z*, together with additional peaks suggesting the presence of multiple isoforms. Such heterogeneity is frequently reported for *Bacillus* lipopeptides and reflects differences in fatty acid chain length, peptide sequence variants, and adduct formation, all of which may influence both physicochemical behavior and biological activity [41]. Although the present data do not allow a definitive structure–activity assignment for each ion detected, the different peak distribution patterns between Solo 1 and Solo 4 suggest that strain-specific isoform composition may contribute to the functional differences observed in surface tension reduction, salinity tolerance, and antimicrobial activity [44,45]. This point is especially important because biosurfactant performance is often discussed generically at the compound-class level, while in practice the exact isoform profile can substantially modulate application-specific properties.

The kinetic results provide another relevant layer of interpretation. Although both strains showed active biomass accumulation during cultivation, their surfactin production profiles differed markedly. Solo 1 reached a maximum surfactin concentration of 90 mg L^−1^ at 24 h, followed by a gradual decline, whereas Solo 4 showed a continuous increase and reached a maximum of 224.4 mg L^−1^ at 72 h. In parallel, surface tension decreased in both strains, confirming the production of extracellular surface-active metabolites. These results indicate that surfactin productivity was not directly proportional to biomass accumulation alone and likely reflected differences in carbon flux distribution, regulatory control of secondary metabolism, nutrient limitation responses, and secretion efficiency [46,47]. In lipopeptide-producing *Bacillus* strains, surfactin synthesis is strongly influenced not only by strain identity but also by physiological state and cultivation conditions, which can explain why strains with different growth and production profiles may show substantial differences in final yield and functional performance [48,49]. From an industrial perspective, this is particularly relevant, since process selection should not rely solely on growth kinetics but rather on the combined assessment of surfactin productivity, surface activity, product stability, and biological functionality.

The physicochemical stability of both biosurfactants is one of the strongest applied findings of this study. Both strains retained activity across a broad temperature interval and remained functional over a pH range of 6–10, while also tolerating salinity conditions up to 5% NaCl. Solo 1 showed greater tolerance under alkaline conditions, whereas Solo 4 maintained more consistent activity under increasing salinity. These results support the idea that the biosurfactants produced by these strains possess the robustness required for processes operating under variable or harsh physicochemical conditions [50]. This is especially important in potential applications such as hydrocarbon-contaminated soil treatment, wastewater remediation, saline or marine systems, and industrial cleaning operations, where fluctuations in pH, ionic strength, and temperature are common [51,52]. In practical terms, a biosurfactant that loses activity under moderate environmental stress has limited technological value, whereas one that preserves surface-active behavior under broad operating ranges is more attractive for scale-up and formulation development [53].

The antimicrobial assays demonstrated that cell-free supernatants from both strains inhibited *S. aureus*, with Solo 4 retaining stronger activity at lower concentrations. This finding extends the functional relevance of these biosurfactants beyond their surface-active properties and suggests that the extracellular fractions contain metabolites with marked antibacterial potential [54]. Recent work has shown that surfactin-containing biosurfactants produced by *Bacillus* strains can inhibit *S. aureus* growth, biofilm formation, and virulence-related traits, supporting their potential as alternative antimicrobial agents [55]. Similarly, Englerová et al. [19] showed that lipopeptide biosurfactants derived from *B. amiloliquefaciencs* reduced *S. aureus* biofilm formation and downregulated biofilm-related genes. In the present study, the stronger antimicrobial profile of Solo 4 is consistent with its higher surfactin yield, greater surface tension reduction, and distinctive MALDI-TOF profile, supporting the idea that quantitative and compositional differences in extracellular metabolites contribute to the functional divergence between the two strains. Similar findings have been reported for antibacterial lipopeptide fractions from *Bacillus* spp., where activity depends not only on the total amount of product but also on the relative abundance of specific isoforms [56]. In addition, the agar diffusion and silicone-tube antibiofilm assays reinforced this trend, confirming the stronger functional performance of Solo 4 under application-oriented conditions.

The evaluation of surfactin-enriched extracts against *Pseudomonas syringae* and *Fusarium oxysporum* broadens the biological relevance of the present study beyond Gram-positive bacterial inhibition. *P. syringae* is an agriculturally relevant Gram-negative phytopathogen, and the inhibition observed in the agar diffusion assay indicates that surfactin-enriched extracts obtained from both strains, particularly Solo 4, retained antibacterial activity against this bacterial target. The activity observed against *P. syringae* is relevant because *Bacillus* cyclic lipopeptides have been increasingly recognized as key metabolites in plant-associated microbial interactions. Recent reviews indicate that surfactin, iturin, and fengycin families contribute not only to direct antagonism against phytopathogens but also to rhizosphere competence, microbial competition, biofilm formation, and plant immune modulation [24,57]. In this context, the inhibition of *P. syringae* by surfactin-enriched extracts, particularly those from Solo 4, supports the potential of these native strains as sources of antimicrobial metabolites with agricultural relevance.

The antifungal assay against *F. oxysporum* further supports the agricultural relevance of the surfactin-enriched extracts. *F. oxysporum* is a major phytopathogenic fungus associated with vascular wilt and root diseases in several crops, and recent studies have shown that *Bacillus*-derived lipopeptides can inhibit *F. oxysporum* through mechanisms involving membrane disruption, hyphal damage, altered spore development, and changes in fungal cell integrity [29,58]. In the present study, Solo 4 produced a larger inhibition zone than Solo 1, indicating stronger antifungal activity. In comparison, copper oxychloride did not produce a visible inhibition zone under the tested conditions. Therefore, these results should be interpreted as an initial indication of antifungal potential that requires validation through additional isolates, dose–response assays, purified fractions, and semi-field or plant-based experiments.

These findings are consistent with recent reports showing that crude or semi-purified lipopeptide fractions from *Bacillus* strains can display antimicrobial activity against phytopathogenic fungi, including *Fusarium* spp. Assena et al. [29] demonstrated that bacterial lipopeptides, including surfactin, iturin, and fengycin-related compounds, contributed to the inhibition of *F. oxysporum* f. sp. *strigae*, affecting fungal development and supporting the potential of lipopeptide-producing bacteria as biological control agents. Similarly, Hussain et al. [30] reported that lipopeptide extracts from *Bacillus subtilis* Sh-17 inhibited *F. oxysporum* f. sp. *lycopersici* and caused structural damage to fungal hyphae and spores. Compared with these studies, the present work contributes additional value by integrating surfactin production kinetics, HPLC quantification, MALDI-TOF profiling, physicochemical stability, antibacterial activity, antifungal activity, and antibiofilm performance within a single comprehensive characterization framework [14,15,16]. This integrated approach strengthens the application-oriented interpretation of Solo 4 as a multifunctional biosurfactant-producing strain.

It is important to note that different biological assays were performed using different sample types. Cell-free supernatants were used for *S. aureus*, whereas surfactin-enriched extracts were evaluated against *P. syringae* and *F. oxysporum*. Therefore, the activity observed against *S. aureus* may reflect the combined action of extracellular metabolites present in the supernatant, while the inhibition observed against *P. syringae* and *F. oxysporum* more directly supports the antimicrobial potential of the surfactin-enriched fraction. This distinction is relevant for interpretation and supports the need for future studies using purified individual isoforms to define structure–activity relationships.

The comparative performance of Solo 1 and Solo 4 suggests that these strains may be better suited to different application niches. Solo 1 showed strong emulsification capacity and greater tolerance at alkaline pH, which may be useful in some environmental or cleaning-related scenarios. In contrast, Solo 4 combined lower final surface tension, higher surfactin yield, better performance under salinity stress, and stronger antimicrobial activity, measurable antifungal activity, and antibiofilm performance, making it a particularly promising candidate for applied biotechnological development. This differentiation is important because it avoids the common oversimplification of treating all *Bacillus*-derived biosurfactants as functionally equivalent. Instead, the present results support a more selective view in which strain identity, metabolite profile, production kinetics, and target organism determine application suitability [45,59,60,61].

While the present study demonstrates the promising biotechnological potential of Solo 1 and Solo 4, further work would strengthen the applied value of these findings. In particular, antimicrobial assays with purified compounds will be useful to define the specific contribution of individual lipopeptides to the activity observed in the cell-free supernatants and extracted fractions. Likewise, although MALDI-TOF/TOF analysis confirmed the presence of multiple lipopeptide isoforms, additional structural characterization would allow more precise homolog assignment and deeper structure–activity interpretation. Finally, evaluation under alternative substrates, broader microbial panels, semi-field conditions, and larger-scale cultivation systems will be important to determine whether the productivity, stability, and antimicrobial performance observed here can be maintained under operational settings relevant to industrial, environmental, biomedical, and agricultural applications [59,60,62].

The combined physicochemical, antibacterial, antifungal, and antibiofilm properties observed in this study significantly strengthen the application-oriented value of these biosurfactants. In particular, the superior performance of Solo 4, characterized by higher surfactin production, stronger surface tension reduction, broader functional stability, and greater inhibitory activity against bacterial and fungal targets, suggests a promising role for this strain in integrated biotechnological applications. These properties are especially relevant in contexts where both surface activity and microbial control are required, such as biofouling prevention, wastewater treatment, surface decontamination, and agricultural pathogen management [61,62].

Although further studies using purified fractions, broader microbial panels, and application-oriented assays will be necessary to determine the specific contribution of individual lipopeptides and optimize performance at larger scales, the present results demonstrate that native rhizospheric *Bacillus* strains from southern Chile constitute a valuable source of multifunctional biosurfactants. Taken together, these findings position Solo 4, in particular, as a promising candidate for the development of sustainable biosurfactant-based systems with potential application in industrial, environmental, biomedical, and agricultural microbial control strategies [59,61,62].

## 4. Materials and Methods

### 4.1. Soil Sampling, Culture Media, and Isolation of Bacterial Strains

Rhizospheric soil samples (500 g) were collected from Lonquimay, La Araucanía Region, Chile (−38.485914, −71.368313). Samples were transported to the laboratory and stored at 4 °C until processing.

Luria-Bertani (LB) medium consisted of 10 g L^−1^ peptone, 5 g L^−1^ yeast extract, and 5 g L^−1^ sodium chloride (NaCl). Mueller–Hinton Broth (MHB) contained 17.5 g L^−1^ hydrolyzed casein, 3.0 g L^−1^ beef extract, and 1.5 g L^−1^ starch. M1 medium consisted of 2.0 g L^−1^ peptone, 4.0 g L^−1^ yeast extract, 10.0 g L^−1^ starch, and 18.0 g L^−1^ agar, adjusted to pH 7.

Bacterial isolation was performed following the methodology described by Lamilla et al. [63], with minor modifications. Briefly, 10 g of soil was suspended in 90 mL of sterile distilled water and agitated at 130 rpm for 10 min. Then, a 1 mL sample was serially diluted up to 10^−4^, and 100 μL of the diluted sample was plated on sterile LB and M1 agar medium. Plates were incubated at 28 °C for 72 h. Distinct colonies were selected based on morphological characteristics and purified by repeated streaking on LB agar plates.

### 4.2. Screening of Biosurfactant-Producing Strains

The selection of biosurfactant-producing strains was carried out based on the presence of complete hemolysis on 5% sheep blood agar, following previously described screening approaches for biosurfactant-producing bacteria [64,65]. Each isolated strain was inoculated onto blood agar plates using a sterile inoculating loop. The plates were incubated at 28 °C for 72 h under controlled temperature conditions. Strains exhibiting clear and transparent halos surrounding the bacterial colonies, indicative of complete hemolysis, were selected for subsequent experimental assays.

### 4.3. Biosurfactant Production and Preliminary Activity Assays

Pure cultures selected based on hemolytic activity were cultivated in LB broth and incubated at 28 °C for 72 h under agitation at 150 rpm. After incubation, biosurfactant production was evaluated. Bacterial cells were recovered by centrifugation at 10,000× *g* for 5 min. Cell-free supernatants were subsequently used for biosurfactant activity assays.

#### 4.3.1. Biosurfactant Production

Selected strains based on hemolytic activity were cultivated in LB broth and incubated at 28 °C for 72 h under agitation at 150 rpm. After incubation, cultures were centrifuged at 10,000× *g* for 5 min to separate the cell-free supernatant. The resulting supernatants were used for oil displacement and emulsification assays using olive oil.

#### 4.3.2. Oil Displacement Assay

Oil displacement activity was evaluated according to the assay described by Morikawa et al. [66], with minor modifications. Oil displacement activity was evaluated by adding 50 mL of distilled water to a 15 cm diameter Petri dish, followed by 20 μL of olive oil onto the water surface. Then, 10 μL aliquots of cell-free supernatants were added. The diameter of the clear zone formed was measured (cm) as an indicator of biosurfactant activity.

#### 4.3.3. Emulsification Index (E24)

The emulsification index (E24) was determined according to Cooper and Goldenberg [67] with minor modifications. First, the emulsifying capacity of the cell-free supernatants obtained from the hemolysis-positive strains was evaluated using olive oil. Equal volumes (2 mL) of cell-free supernatants and hydrophobic substrate were mixed and vortexed for 2 min. The mixtures were left undisturbed at room temperature for 24 h. The emulsification index (E24) was calculated by dividing the height of the emulsified layer (“he”) by the total height (“ht”) and multiplying by 100 to obtain the percentage. Based on this preliminary E24 screening, together with oil displacement activity, strains Solo 1 and Solo 4 were selected for subsequent characterization.

To broaden the evaluation of emulsifying capacity, cell-free supernatants from strains Solo 1 and Solo 4 were subsequently tested against different water-immiscible substrates, including recycled cooking oil, motor oil, corn oil, and n-hexadecane. The same E24 procedure was applied for each substrate. All assays were performed in triplicate, and results are expressed as mean ± standard deviation.

$$E24\%=\frac{he}{ht}\times 100$$

### 4.4. Fermentation Conditions and Kinetic Monitoring

Fermentation assays were performed using the selected biosurfactant-producing strains Solo 1 and Solo 4. Pre-inocula were prepared in nutrient broth supplemented with 0.5% (*w*/*v*) yeast extract and incubated at 28 °C for 8–12 h until the exponential growth phase was reached. The cultures were then used to inoculate 500 mL Erlenmeyer flasks containing 100 mL of LB medium at 2% (*v*/*v*). Fermentations were carried out at 28 °C under agitation at 150 rpm for 72 h. Samples were collected at defined time points during cultivation to evaluate biomass concentration, surface tension reduction, and surfactin concentration. Bacterial cells were separated from culture broth by centrifugation at 10,000× *g* for 10 min at 4 °C, and the resulting cell-free supernatants were used for surface tension measurements and surfactin extraction/quantification.

### 4.5. Extraction of Surfactin-Enriched Lipopeptide Fractions

Surfactin-enriched lipopeptide fractions were recovered from fermentation broths following an acid precipitation procedure. Cultures of strains Solo 1 and Solo 4 were centrifuged at 8000× *g* for 20 min to remove bacterial cells and obtain cell-free supernatants based on the methodology described by Hu et al. [68]. The supernatants were acidified to pH 2.0 using 2 N HCl and maintained overnight at 4 °C to promote lipopeptide precipitation. The precipitated material was recovered by centrifugation at 8000× *g* for 20 min, dried at 37 °C for 48 h, and resuspended in 2 mL of methanol. The resulting extracts were filtered through 0.22 μm membranes prior to HPLC quantification and MALDI-TOF/TOF analysis.

### 4.6. Molecular Identification of Selected Strains

Genomic DNA was extracted from the selected bacterial strains using the DNeasy UltraClean^®^ Microbial Kit (Qiagen, Hilden, Germany), following the manufacturer’s instructions. The 16S rRNA gene was amplified by polymerase chain reaction (PCR). Each PCR reaction mixture (50 μL) contained 100 ng of genomic DNA, 1× GoTaq^®^ Green Master Mix (Promega, Madison, WI, USA), and 0.2 μM of the universal primers 27F (5′-AGAGTTTGATCMTGGCTCAG-3′) and 1492R (5′-TACGGYTACCTTGTTACGACTT-3′). Nuclease-free water was used to adjust the final reaction volume. PCR amplification was performed under the following conditions: initial denaturation at 94 °C for 5 min; 30 cycles of denaturation at 94 °C for 30 s, annealing at 50 °C for 90 s, and extension at 72 °C for 90 s; followed by a final extension at 72 °C for 10 min. The amplified 16S rRNA gene fragments (~1.5 kb) were visualized by electrophoresis on a 1.5% (*w*/*v*) agarose gel stained with GelRed^®^ 3× (Merck Millipore, Temecula, CA, USA). PCR products were sequenced using a 3500 Genetic Analyzer (Applied Biosystems, Foster City, CA, USA) at Bioren (Chile). Sequence similarity searches were performed using the BLASTn algorithm against the GenBank database (NCBI, Bethesda, MD, USA). Phylogenetic analysis was conducted using MEGA11: Molecular Evolutionary Genetics Analysis version 11. software, incorporating representative sequences retrieved from GenBank. The reliability of the phylogenetic tree was assessed by bootstrap analysis with 1000 replications.

### 4.7. Detection of the Surfactin Biosynthesis Gene (srfAA)

Genomic DNA was extracted using the UltraClean^®^ Microbial DNA Isolation Kit (MO BIO Laboratories, Carlsbad, CA, USA) according to the manufacturer’s instructions. The gene surfactin (srfAA) was selectively amplified from genomic DNA by polymerase chain reaction (PCR) using primers F-5 TCGGGACAGGAAGACATCAT 3″ and R 5′ CCACTCAAACGGATAATCCTGA 3′, enabling the amplification of approximately 201 bp. PCR amplification was performed in a Multigene Optimax Thermal Cycler (Labnet International, Edison, NJ, USA) in 50 μL of PCR mix comprising 25 μL of mix reaction buffer 2x (SapphireAmp Fast PCR Master Mix, Takara Bio Inc., Kusatsu, Shiga, Japan), 22 μL of ultra-pure water, 1 μL of each primer (10 μM), and 5 μL of DNA. The PCR amplifications were performed using the following cycle conditions: initial activation at 95 °C for 4 min; 35 cycles of 94 °C for 1 min, followed by annealing for 30 s at different temperatures depending on the primers used, an extension step of 1 min at 70 °C, and a final extension step of 5 min at 70 °C. The experiment included a negative control mixture without added DNA. The amplification reaction was analyzed by electrophoresis using a 1% agarose gel, followed by staining with Gel Red.

### 4.8. HPLC Quantification of Surfactin

Surfactin concentration was determined by reversed-phase high-performance liquid chromatography (RP-HPLC). Chromatographic separation was performed using a Merck Hitachi L-2130 pump (Hitachi High-Technologies Corporation, Tokyo, Japan) coupled to a Rheodyne 7725 manual injector (Rheodyne, Rohnert Park, CA, USA) and a Merck Hitachi L-2455 diode array detector (Hitachi High-Technologies Corporation, Tokyo, Japan). Separation was achieved on a Chromolith^®^ RP-18e C18 column (Merck KGaA, Darmstadt, Germany; 4.6 mm × 100 mm). The mobile phase consisted of solvent A, ultrapure water containing 0.1% (*v*/*v*) trifluoroacetic acid (TFA), and solvent B, acetonitrile containing 0.1% (*v*/*v*) TFA. Elution was carried out using a linear gradient from 20% to 80% solvent B over 30 min, followed by an isocratic step at 80% solvent B for 5 min. The flow rate was 1.0 mL min^−1^, the column temperature was maintained at 30 °C, and the injection volume was 20 µL. Surfactin was detected at 210 nm. Identification was based on comparison of retention times and UV spectra with a commercial surfactin standard (≥98% purity; Sigma-Aldrich, São Paulo, Brazil). Quantification was performed using an external calibration curve prepared from serial dilutions of a surfactin stock solution at 2500 mg L^−1^, and results are expressed as mg L^−1^.

### 4.9. MALDI-TOF/TOF Analysis

Biosurfactants were chemically characterized and quantified using matrix-assisted laser desorption/ionization time-of-flight mass spectrometry (MALDI-TOF/TOF) and high-performance liquid chromatography (HPLC). MALDI-TOF mass spectrometric analyses were performed using a Micromass TOFSpec 2E Time-of-Flight Mass Spectrometer (Micromass/Waters, Manchester, UK) equipped with a nitrogen laser operating at a wavelength of 337 nm and a repetition rate of 5 Hz, as well as a time-lag focusing unit. Ions were generated by laser irradiation at energies slightly above the ionization threshold. Positive-ion spectra were acquired in reflectron mode using an accelerating voltage of 20 kV and were externally calibrated with a suitable mixture of poly(ethylene glycol) (PEG) standards. For each spectrum, 100–150 laser shots were accumulated to improve the signal-to-noise ratio. Data acquisition and processing were performed using MassLynx software version 3.5 (Micromass/Waters, Manchester, UK).

Sample preparation for MALDI-TOF analysis was carried out by mixing a dithranol matrix solution (10 mg/mL in tetrahydrofuran, THF), the biosurfactant analyte solution (2 mg/mL in THF), and a sodium trifluoroacetate solution (CF_3_COONa, NaTFA; 0.1 mg/mL in THF) in a volume ratio of 7:1:1 (*v*/*v*/*v*). Subsequently, 0.5 μL of the resulting mixture was deposited onto a stainless-steel MALDI target plate and allowed to air-dry prior to analysis.

### 4.10. Stability Assays of Biosurfactants

The stability of biosurfactant was assessed under different temperature, pH, and salinity conditions following the approach described by Wu et al. [40], with modifications. Thermal stability was evaluated by exposing biosurfactant samples to temperatures ranging from 4 to 121 °C for 30 min, followed by cooling to room temperature. pH stability was evaluated over a pH range of 1–10, adjusted with 1 N HCl or 1 N NaOH. Salinity stability was assessed using NaCl concentrations ranging from 1% to 20% (*w*/*v*). After each treatment, residual biosurfactant activity was evaluated by surface tension measurements and/or emulsification index (E24), according to the corresponding assay conditions.

### 4.11. Antibacterial and Antibiofilm Activity of Cell-Free Supernatants Against Staphylococcus aureus

#### 4.11.1. Growth Inhibition Assay

Cell-free supernatants from Solo 1 and Solo 4 strains were evaluated against a pathogenic bacterial strain. For this purpose, *Staphylococcus aureus* ATCC 6358 was selected as a representative of the Gram-positive and biofilm-producing group. The culture was maintained according to the prescribed details, and the assay was performed as described by Stepanovic et al. [69]. Log-phase cells were prepared and exposed to different concentrations of cell-free supernatants (100, 75, 25, 10, and 0%). After incubation at 37 °C for 24 h, bacterial growth was quantified by measuring optical density at 600 nm (OD600). Growth inhibition (%) was calculated relative to the untreated control. All assays were performed in triplicate, and results are expressed as mean ± standard deviation.

#### 4.11.2. Antibacterial Activity by Agar Diffusion Assay

The antimicrobial activity of biosurfactant samples obtained from strains Solo 1 and Solo 4 was also evaluated using an agar diffusion assay against *S. aureus* following previously described procedures [70], with minor modifications. Bacterial cultures were prepared on LB agar plates for 24 h at 37 °C. A bacterial suspension was prepared in sterile 0.9% (*w*/*v*) saline solution and uniformly spread onto Mueller–Hinton agar plates. Discs containing biosurfactant samples at concentrations of 25 and 100 mg L^−1^ were placed on the agar surface. Florfenicol (8000 mg L^−1^) was used as a positive control. Plates were incubated at 37 °C for 24 h, and inhibition zones were measured in millimeters (mm) as the average of two perpendicular diameters. All assays were performed in triplicate.

#### 4.11.3. Antibiofilm Activity on Silicone Tubes

Antibiofilm activity was evaluated using sterile silicone tubes as abiotic surfaces adapted from previously described assays [70]. Briefly, 10 μL of overnight *S. aureus* culture was inoculated into 5 mL of fresh LB medium. Subsequently, 1000 μL of biosurfactant solution at 100 mg L^−1^ was added to the system. Sterile silicone tubes (4 cm in length) were immersed in the inoculated medium and incubated at 37 °C for 24 h under static conditions. After incubation, the silicone tubes were washed twice with sterile distilled water to remove planktonic and loosely attached cells, air-dried, and stained with 0.1% (*w*/*v*) crystal violet for 20 min. Excess dye was removed by washing with distilled water, and the stained tubes were air-dried for 30 min at room temperature.

Biofilm formation was evaluated using a semi-quantitative scoring system ranging from 0 to 4, where 0 indicated no biofilm formation, and 4 indicated abundant biofilm formation. The percentage of inhibition was calculated as follows:

$$\%\;inhibition\;=\frac{\left(Score\;control-Score\;treatment\right)}{\left(Score\;control\right)}\times \;100$$

### 4.12. Antimicrobial Activity of Surfactin-Enriched Extracts Against Phytopathogens

#### 4.12.1. Antibacterial Activity Against *Pseudomonas syringae*

The antibacterial activity of surfactin-enriched extracts from strains Solo 1 and Solo 4 was evaluated against *Pseudomonas syringae* as a representative Gram-negative phytopathogenic bacterium using an agar diffusion assay, adapted from previously described studies [70]. Bacterial cultures were grown on LB agar plates at 37 °C for 24 h. A bacterial suspension was uniformly spread onto agar plates, and surfactin-enriched extracts at different concentrations (100, 75, 25, 10, and 0%) were applied onto the agar surface. Florfenicol (8000 mg L^−1^) was used as a positive control. Antibacterial activity was determined by measuring the diameter of the inhibition zones (mm). Relative antibacterial activity (%) was calculated using the antibiotic control as 100%. All assays were performed in triplicate, and results are expressed as mean ± standard deviation.

#### 4.12.2. Antifungal Activity Against *Fusarium oxysporum*

The antifungal activity of surfactin-enriched extracts from Solo 1 and Solo 4 was evaluated against *Fusarium oxysporum* using the mycelial growth inhibition assay on potato dextrose agar (PDA) adapted from Assena et al. [29]. A 0.5 cm diameter mycelial disc from a seven-day-old fungal culture was placed at the center of each Petri dish containing 30 mL of PDA medium. Five equidistant wells were made in the agar, and 100 μL of surfactin-enriched extract (100 mg L^−1^) was added to two of the wells. Copper oxychloride at 10,000 mg L^−1^ was used as a fungicide reference treatment. Plates were incubated at 25 °C for three days, and antifungal activity was assessed by measuring the inhibition zone surrounding each treatment well. Results are expressed as inhibition zone diameter (mm).

### 4.13. Control Experiments, Method Validation, and Statistical Analysis

Control treatments were included in all antimicrobial, antibiofilm, and antifungal assays. Untreated bacterial cultures were used as negative controls, whereas florfenicol (8000 mg L^−1^) was used as the positive antibacterial control in agar diffusion assays against *S. aureus* and *P. syringae*. In the antibiofilm assay, untreated silicone tubes inoculated with *S. aureus* were used as biofilm-forming controls, while tubes treated with the cell-free supernatant from Solo 4 were used as treated samples. For the antifungal assay, copper oxychloride (10,000 mg L^−1^) was included as a fungicide reference treatment; however, no visible inhibition zone was observed under the evaluated conditions.

All assays were performed in duplicate or triplicate, as indicated for each experiment. Data are presented as mean ± standard deviation. Inhibition zones were measured as the average of two perpendicular diameters. Relative antibacterial activity was calculated using florfenicol as 100%. Biofilm inhibition was calculated relative to the untreated control using a semi-quantitative 0–4 scale.

Statistical analyses were performed using GraphPad Prism version 8.0. When applicable, data were analyzed by one-way ANOVA followed by Tukey’s multiple-comparison test. Differences were considered statistically significant at *p* < 0.05.

## 5. Conclusions

This study comprehensively demonstrated that rhizospheric soils from La Araucanía, Chile, constitute a valuable biological source of biosurfactant-producing bacteria. Among 18 isolated strains, two high-performing isolates, Solo 1 and Solo 4, identified as *Bacillus amyloliquefaciens* and *Bacillus subtilis*, respectively, exhibited strong biosurfactant production capacity. Both strains harbored the surfactin biosynthesis gene (*srfAA)* and produced distinct lipopeptide isoforms, which were confirmed by MALDI-TOF/TOF MS, revealing multiple isoforms, and further quantified by HPLC, which showed distinct production profiles between both strains.

Kinetic analysis indicated that Solo 1 reached an early production peak, whereas Solo 4 exhibited sustained surfactin production and achieved a higher final surfactin concentration. Both biosurfactants showed remarkable stability across a wide range of temperature, pH, and salinity conditions, maintaining surface activity properties under environmentally relevant conditions.

The antimicrobial and antibiofilm assays expanded the functional relevance of these biosurfactants. Cell-free supernatants from both strains inhibited the growth of *Staphylococcus aureus*, with Solo 4 consistently demonstrating stronger antibacterial activity and pronounced antibiofilm activity on silicone surfaces. In addition, surfactin-enriched extracts showed inhibitory activity against *Pseudomonas syringae* and the filamentous fungus *Fusarium oxysporum,* further supporting the broader antimicrobial potential of these lipopeptide fractions.

Taken together, these findings highlight the multifunctional nature of *Bacillus*-derived biosurfactants, combining physicochemical performance with antibacterial, antifungal, and antibiofilm activity. In particular, the superior performance of *Bacillus subtilis* Solo 4 suggests its potential as a promising candidate for applications requiring both surface-active and microbial control properties. These may include biofouling control, surface sanitation, wastewater treatment, agricultural pathogen management, and relevant biotechnological processes.

Nevertheless, this study has some limitations that should be addressed in future work. The antimicrobial assays were performed under in vitro conditions and using different sample types, including cell-free supernatants and surfactin-enriched extracts. Therefore, the specific contribution of individual lipopeptide isoforms remains to be determined. Future studies should include purified surfactin homologues, broader microbial panels, dose–response assays, phytotoxicity and cytotoxicity evaluations, greenhouse or semi-field experiments, and scale-up studies using low-cost substrates.

Overall, the superior performance of Solo 4 suggests that this native *Bacillus subtilis* strain is a promising candidate for the development of biosurfactant-based systems with potential applications in antimicrobial control, biofilm management, surface sanitation, wastewater treatment, and agricultural pathogen suppression.

## Figures and Tables

**Table 1:** Morphological characteristics of bacterial colonies of isolated strains and selection of biosurfactant-producing strains.

Strain	Color	Hemolysis	Strain	Color	Hemolysis
Solo 1	Cream	Positive	Solo 10	White	Positive
Solo 2	White	Negative	Solo 11	White	Negative
Solo 3	White	Positive	Solo 12	Gray	Positive
Solo 4	White	Positive	Solo 13	Cream	Negative
Solo 5	White	Positive	Solo 14	White	Negative
Solo 6	White	Negative	Solo 15	Orange	Negative
Solo 7	Yellow	Negative	Solo 16	Cream	Negative
Solo 8	White	Negative	Solo 17	Cream	Negative
Solo 9	Orange	Negative	Solo 18	Cream	Negative

**Table 2:** Antibacterial activity of biosurfactant samples from strains Solo 1 and Solo 4 against *Staphylococcus aureus* determined by agar diffusion assay.

Treatment	Concentration (mg L^−1^)	Halo (mm)	Relative Activity (%)
Control	8000	40 ± 0.5	100
Solo 1	25	12 ± 0.7	30
Solo 1	100	20 ± 0.25	50
Solo 4	25	18 ± 0.5	45
Solo 4	100	30 ± 0.6	75

**Table 3:** Semi-quantitative evaluation of biofilm formation of *Staphylococcus aureus* on silicone surfaces under control conditions and after treatment with cell-free supernatants from strain Solo 4.

Treatment	Rep 1	Rep 2	Rep 3	Mean
Control	++++	++++	+++	3.7
Solo 4	+	+	-	0.7

Note: Biofilm formation was evaluated using a semi-quantitative scoring scale based on crystal violet staining intensity: −, no visible biofilm formation; +, weak biofilm formation; ++, moderate biofilm formation; +++, strong biofilm formation; ++++, very strong biofilm formation.

**Table 4:** Antibacterial activity of surfactin-enriched extracts from strains Solo 1 and Solo 4 against *Pseudomonas syringae* determined by agar diffusion assay.

Treatment	Extract Concentration (%)	Halo (mm)	Relative Activity (%)
Solo 1	100	15.9 ± 2.0	79.5
Solo 1	75	10.0 ± 3.0	50
Solo 1	25	7.0 ± 3.0	35
Solo 1	10	0 ± 0	0
Control	8000 mg L^−1^	20.0 ± 1.0	100
Solo 4	100	18.0 ± 3.0	90
Solo 4	75	15.0 ± 1.0	75
Solo 4	25	8.0 ± 2.0	38
Solo 4	10	4.0 ± 1.0	20

**Table 5:** Antifungal activity of surfactin-enriched extracts from strains Solo 1 and Solo 4 against *Fusarium oxysporum* determined by agar diffusion assay.

Treatment	Concentration (mg L^−1^)	Inhibition Zone (mm)
Copper oxychloride	10,000	0.0 ± 0.0
Solo 1	100	5.2 ± 0.3
Solo 4	100	15.29 ± 0.6

Note: Copper oxychloride did not produce a visible inhibition zone under the evaluated conditions.

## Data Availability

Data are contained within the article.

## References

[1] Burgos-Díaz C., Pons R., Espuny M.J., Aranda F.J., Teruel J.A., Manresa A., Ortiz A., Marqués A.M. (2011). Partial Characterization of a Biosurfactant Mixture Produced by *Sphingobacterium* sp. Isolated from Soil. J. Colloid Interface Sci..

[2] Ebnesajjad S., Landrock A.H. (2015). Surface Tension and Its Measurement. Adhesives Technology Handbook.

[3] Ambaye T.G., Vaccari M., Prasad S., Rtimi S. (2021). Preparation, Characterization and Application of Biosurfactant in Various Industries: A Critical Review on Progress, Challenges and Perspectives. Environ. Technol. Innov..

[4] Ambaye T.G., Formicola F., Sbaffoni S., Prasad S., Milanese C., Cuna F.S.R.d., Franzetti A., Vaccari M. (2023). Treatment of Petroleum Hydrocarbon Contaminated Soil by Combination of Electro-Fenton and Biosurfactant-Assisted Bioslurry Process. Chemosphere.

[5] Ammami M.T., Portet-Koltalo F., Benamar A., Duclairoir-Poc C., Wang H., Le Derf F. (2015). Application of Biosurfactants and Periodic Voltage Gradient for Enhanced Electrokinetic Remediation of Metals and PAHs in Dredged Marine Sediments. Chemosphere.

[6] Malakar C., Ali M., Patowary R., Deka S. (2024). Production of Lipopeptide Biosurfactant Using Wastewater from Parboiled Paddy Rice and Evaluation of Antifungal Property of the Biosurfactant Against Two Dermatophyte Fungi. Appl. Biochem. Biotechnol..

[7] Omore I.A., Yusuf I., Ochayi H.E. (2024). Isolation of Biosurfactant-Producing and Crude Oil-Degrading Bacterium, *Enterococcus hirae*, from Hydrocarbon-Polluted Soils and Characterization of the Biosurfactant Produced. Biol. Environ. Sci. J. Trop..

[8] Lima B.G.A., Silva R.R., Meira H.M., Durval I.J.B., Macedo Bezerra Filho C., Silva T.A.L., Sarubbo L.A., Luna J.M. (2024). Synthesis and Characterization of Silver Nanoparticles Stabilized with Biosurfactant and Application as an Antimicrobial Agent. Microorganisms.

[9] Sarubbo L., Silva M., Durval I., Bezerra K., Ribeiro B., Silva I., Twigg M., Banat I. (2022). Biosurfactants: Production, Properties, Applications, Trends, and General Perspectives. Biochem. Eng. J..

[10] Abu-Ruwaida A.S., Banat I.M., Haditirto S., Salem A., Kadri M. (1991). Isolation of Biosurfactant-Producing Bacteria Product Characterization, and Evaluation. Acta Biotechnol..

[11] Banat I.M., Franzetti A., Gandolfi I., Bestetti G., Martinotti M.G., Fracchia L., Smyth T.J., Marchant R. (2010). Microbial Biosurfactants Production, Applications and Future Potential. Appl. Microbiol. Biotechnol..

[12] Akbari S., Abdurahman N.H., Yunus R.M., Fayaz F., Alara O.R. (2018). Biosurfactants—A New Frontier for Social and Environmental Safety: A Mini Review. Biotechnol. Res. Innov..

[13] Gayathiri E., Prakash P., Karmegam N., Varjani S., Awasthi M.K., Ravindran B. (2022). Biosurfactants: Potential and Eco-Friendly Material for Sustainable Agriculture and Environmental Safety—A Review. Agronomy.

[14] Sharma N., Lavania M., Lal B. (2022). Biosurfactant: A Next-Generation Tool for Sustainable Remediation of Organic Pollutants. Front. Microbiol..

[15] Miao Y., To M.H., Siddiqui M.A., Wang H., Lodens S., Chopra S.S., Kaur G., Roelants S.L.K.W., Lin C.S.K. (2024). Sustainable biosurfactant production from secondary feedstock—Recent advances, process optimization and perspectives. Front. Chem..

[16] Mulligan C.N. (2021). Sustainable Remediation of Contaminated Soil Using Biosurfactants. Front. Bioeng. Biotechnol..

[17] Alajlani M., Shiekh A., Hasnain S., Brantner A. (2016). Purification of Bioactive Lipopeptides Produced by *Bacillus subtilis* Strain BIA. Chromatographia.

[18] Gerber S., Wulf M., Milkereit G., Vill V., Howe J., Roessle M., Garidel P., Gutsmann T., Brandenburg K. (2009). Phase Diagrams of Monoacylated Amide-Linked Disaccharide Glycolipids. Chem. Phys. Lipids.

[19] Englerová K., Bedlovičová Z., Nemcová R., Király J., Maďar M., Hajdučková V., Styková E., Mucha R., Reiffová K. (2021). *Bacillus amyloliquefaciens*—Derived Lipopeptide Biosurfactants Inhibit Biofilm Formation and Expression of Biofilm-Related Genes of *Staphylococcus aureus*. Antibiotics.

[20] Karamchandani B., Pawar A., Pawar S., Syed S., Mone N., Dalvi S., Rahman P., Banat I., Satpute S. (2022). Biosurfactants’ multifarious functional potential for sustainable agricultural practices. Front. Bioeng. Biotechnol..

[21] Yaraguppi D., Bagewadi Z., Patil N., Mantri N. (2023). Iturin: A Promising Cyclic Lipopeptide with Diverse Applications. Biomolecules.

[22] Markelova N., Chumak A. (2025). Antimicrobial Activity of *Bacillus* Cyclic Lipopeptides and Their Role in the Host Adaptive Response to Changes in Environmental Conditions. Int. J. Mol. Sci..

[23] Karnwal A. (2023). Prospects of Microbial Bio-surfactants to Endorse Prolonged Conservation in the Pharmaceutical and Agriculture Industries. Chem. Sel..

[24] Reddy A.S., Chen C.Y., Baker S.C., Chen C.C., Jean J.S., Fan C.W., Chen H.R., Wang J.C. (2009). Synthesis of silver nanoparticles using surfactin: A biosurfactant as stabilizing agent. Mater. Lett..

[25] Ceresa C., Fracchia L., Fedeli E., Porta C., Banat I. (2021). Recent Advances in Biomedical, Therapeutic and Pharmaceutical Applications of Microbial Surfactants. Pharmaceutics.

[26] Zhen C., Ge X., Lu Y., Liu W. (2023). Chemical structure, properties and potential applications of surfactin, as well as advanced strategies for improving its microbial production. AIMS Microbiol..

[27] Balleux G., Höfte M., Arguelles-Arias A., Deleu M., Ongena M. (2025). *Bacillus* lipopeptides as key players in rhizosphere chemical ecology. Trends Microbiol..

[28] Ding N., Dong H., Ongena M. (2025). Bacterial cyclic lipopeptides as triggers of plant immunity and systemic resistance against pathogens. Plants.

[29] Assena M., Pfannstiel J., Rasche F. (2024). Inhibitory activity of bacterial lipopeptides against *Fusarium oxysporum* f. sp. *strigae*. BMC Microbiol..

[30] Hussain S., Ali M., Ghazy A., Al-Doss A., Attia K., Shah T., Li F. (2025). Identification of antifungal lipopeptides from *Bacillus subtilis* Sh-17 targeting *Fusarium oxysporum* f. sp. *lycopersici*. Chem. Biol. Technol. Agric..

[31] Khademolhosseini R., Jafari A., Mousavi S., Hajfarajollah H., Noghabi K., Manteghian M. (2019). Physicochemical characterization and optimization of glycolipid biosurfactant production by a native strain of Pseudomonas aeruginosa HAK01 and its performance evaluation for the MEOR process. RSC Adv..

[32] Biktasheva L., Gordeev A., Kirichenko A., Kuryntseva P., Selivanovskaya S. (2024). Screening of Microorganisms from Wastes and Identification of the Optimal Substrate for Biosurfactant Production. Microbiol. Res..

[33] Fardami A.Y., Kawo A.H., Yahaya S., Lawal I., Abubakar A.S., Maiyadi K.A. (2022). A Review On Biosurfactant Properties, Production And Producing Microorganisms. J. Biochem. Microbiol. Biotechnol..

[34] Shahid I., Han J., Hanooq S., Malik K., Borchers C., Mehnaz S. (2021). Profiling of Metabolites of *Bacillus* spp. and Their Application in Sustainable Plant Growth Promotion and Biocontrol. Front. Sustain. Food Syst..

[35] Zhang N., Wang Z., Shao J., Xu Z., Liu Y., Xun W., Miao Y., Shen Q., Zhang R. (2023). Biocontrol mechanisms of *Bacillus*: Improving the efficiency of green agriculture. Microb. Biotechnol..

[36] Andrić S., Meyer T., Ongena M. (2020). *Bacillus* Responses to Plant-Associated Fungal and Bacterial Communities. Front. Microbiol..

[37] Liu Y., Xu Z., Chen L., Xun W., Shu X., Chen Y., Sun X., Wang Z., Ren Y., Shen Q. (2024). Root colonization by beneficial rhizobacteria. FEMS Microbiol. Rev..

[38] Diez M.C., Llafquén C., Fincheira P., Lamilla C., Briceño G., Schalchli H. (2022). Biosurfactant Production by *Bacillus amyloliquefaciens* C11 and *Streptomyces lavendulae* C27 Isolated from a Biopurification System for Environmental Applications. Microorganisms.

[39] Dini S., Bekhit A., Roohinejad S., Vale J., Agyei D. (2024). The Physicochemical and Functional Properties of Biosurfactants: A Review. Molecules.

[40] Wu B., Xiu J., Yu L., Huang L., Yi L., Ma Y. (2022). Biosurfactant production by *Bacillus subtilis* SL and its potential for enhanced oil recovery in low permeability reservoirs. Sci. Rep..

[41] Théatre A., Cano-Prieto C., Bartolini M., Laurin Y., Deleu M., Niehren J., Fida T., Gerbinet S., Alanjary M., Medema M.H. (2021). The Surfactin-Like Lipopeptides From *Bacillus* spp.: Natural Biodiversity and Synthetic Biology for a Broader Application Range. Front. Bioeng. Biotechnol..

[42] Qiao J., Borriss R., Sun K., Zhang R., Chen X., Liu Y., Liu Y. (2024). Research advances in the identification of regulatory mechanisms of surfactin production by *Bacillus*: A review. Microb. Cell Fact..

[43] Guo Z., Sun J., Ma Q., Li M., Dou Y., Yang S., Gao X. (2024). Improving Surfactin Production in *Bacillus subtilis* 168 by Metabolic Engineering. Microorganisms.

[44] Grifé-Ruiz M., Hierrezuelo-León J., De Vicente A., Pérez-García A., Romero D. (2025). Diversification of Lipopeptide Analogues Drives Versatility in Biological Activities. J. Agric. Food Chem..

[45] Ma Y., Kong Q., Qin C., Chen Y., Chen Y., Lv R., Zhou G. (2016). Identification of lipopeptides in *Bacillus megaterium* by two-step ultrafiltration and LC–ESI–MS/MS. AMB Expr..

[46] Hiller E., Off M., Hermann A., Vahidinasab M., Perino E., Lilge L., Hausmann R. (2024). The influence of growth rate-controlling feeding strategy on the surfactin production in *Bacillus subtilis* bioreactor processes. Microb. Cell Fact..

[47] Liu T., Zheng Y., Wang L., Wang X., Wang H., Tian Y. (2025). Optimizing surfactin yield in *Bacillus velezensis* BN to enhance biocontrol efficacy and rhizosphere colonization. Front. Microbiol..

[48] Bartal A., Vigneshwari A., Bóka B., Vörös M., Takács I., Kredics L., Manczinger L., Varga M., Vágvölgyi C., Szekeres A. (2018). Effects of Different Cultivation Parameters on the Production of Surfactin Variants by a *Bacillus subtilis* Strain. Molecules.

[49] Geissler M., Kühle I., Heravi K., Altenbuchner J., Henkel M., Hausmann R. (2019). Evaluation of surfactin synthesis in a genome reduced *Bacillus subtilis* strain. AMB Express.

[50] Eras-Muñoz E., Farré A., Sánchez A., Font X., Gea T. (2022). Microbial biosurfactants: A review of recent environmental applications. Bioengineered.

[51] Ali F., Das S., Hossain T., Chowdhury S., Zedny S., Das T., Chowdhury M., Uddin M. (2021). Production optimization, stability and oil emulsifying potential of biosurfactants from selected bacteria isolated from oil-contaminated sites. R. Soc. Open Sci..

[52] Filho A., Converti A., Da Silva R., Sarubbo L. (2023). Biosurfactants as Multifunctional Remediation Agents of Environmental Pollutants Generated by the Petroleum Industry. Energies.

[53] Ibrahim S., Khalil K., Zahri K., Gomez-Fuentes C., Convey P., Zulkharnain A., Sabri S., Alias S., González-Rocha G., Ahmad S. (2023). Biosurfactant Production and Growth Kinetics Studies of the Waste Canola Oil-Degrading Bacterium *Rhodococcus erythropolis* AQ5-07 from Antarctica. Molecules.

[54] Jeong G., Kim D., Cho K., Choi E., Jung W., Khan F., Kim Y. (2025). Surface and Functional Properties of Biosurfactant Produced by *Bacillus rugosus* HH2 Derived from Jeotgal. J. Microbiol. Biotechnol..

[55] Jeong G., Kim D., Park D., Cho K., Kim M., Oh D., Tabassum N., Jung W., Khan F., Kim Y. (2024). Control of *Staphylococcus aureus* infection by biosurfactant derived from *Bacillus rugosus* HH2: Strain isolation, structural characterization, and mechanistic insights. J. Hazard. Mater..

[56] Myo N., Kamwa R., Jamnong T., Swasdipisal B., Somrak P., Rattanamalakorn P., Neatsawang V., Apiwatsiri P., Yata T., Hampson D. (2025). Metabolomic profiling and antibacterial efficacy of probiotic-derived cell-free supernatant encapsulated in nanostructured lipid carriers against canine multidrug-resistant bacteria. Front. Vet. Sci..

[57] Valenzuela Ruiz V., Gándara-Ledezma A., Villarreal-Delgado M.F., Villa-Rodríguez E.D., Parra-Cota F.I., Santoyo G., Gómez-Godínez L.J., Cira Chávez L.A., de los Santos-Villalobos S. (2024). Regulation, Biosynthesis, and Extraction of *Bacillus*-Derived Lipopeptides and Its Implications in Biological Control of Phytopathogens. Stresses.

[58] Pereira J., Gudiña E., Costa R., Vitorino R., Teixeira J., Coutinho J., Rodrigues L. (2013). Optimization and characterization of biosurfactant production by *Bacillus subtilis* isolates towards microbial enhanced oil recovery applications. Fuel.

[59] Mnif I., Ghribi D. (2015). Review lipopeptides biosurfactants: Mean classes and new insights for industrial, biomedical, and environmental applications. Biopolymers.

[60] Valdés-Velasco L.M., Favela-Torres E., Théatre A., Arguelles-Arias A., Saucedo-Castañeda G., Jacques P., Saucedo-Castañeda G. (2022). Relationship between lipopeptide biosurfactant and primary metabolite production by *Bacillus* strains in solid-state and submerged fermentation. Bioresour. Technol..

[61] Jimoh A., Booysen E., van Zyl L., Trindade M. (2023). Do biosurfactants as anti-biofilm agents have a future in industrial water systems?. Front. Bioeng. Biotechnol..

[62] Saiyam D., Dubey A., Malla M.A., Kumar A. (2024). Lipopeptides from *Bacillus*: Unveiling biotechnological prospects—Sources, properties, and diverse applications. Braz. J. Microbiol..

[63] Carrillo P.G., Mardaraz C., Pitta-Alvarez S.I., Giulietti A.M. (1996). Isolation and selection of biosurfactant-producing bacteria. World J. Microbiol. Biotechnol..

[64] Youssef N.H., Duncan K.E., Nagle D.P., Savage K.N., Knapp R.M., McInerney M.J. (2004). Comparison of methods to detect biosurfactant production by diverse microorganisms. J. Microbiol. Methods.

[65] Lamilla C., Braga D., Castro R., Guimaraes C., de Castilho L., Freire D., Barrientos L. (2018). *Streptomyces luridus* So3.2 from Antartic soil as a novel produces of compounds with bioemulsification potential. PLoS ONE.

[66] Morikawa M., Hirata Y., Imanaka T. (2000). A study on the structure–function relationship of lipopeptide biosurfactants. Biochim. Biophys. Acta (BBA) Mol. Cell Biol. Lipids.

[67] Cooper D.G., Goldenberg B.G. (1987). Surface-active agents from two *Bacillus* species. Appl. Environ. Microbiol..

[68] Hu M., Yu J., Zhang H., Xu Q. (2022). An efficient method for the recovery and separation of surfactin from fermentation broth by extraction-back extraction. Process Biochem..

[69] Stepanovic S., Vukovic D., Dakic I., Savic B., Svabic-Vlahovic M. (2000). A modified microtiter-plate test for quantification of staphylococcal biofilm formation. J. Microbiol. Methods.

[70] Thakur B., Pathak V.M., Kumar V., Rathour R.K. (2024). Unveiling the antimicrobial and antibiofilm potential of biosurfactant produced by newly isolated *Lactiplantibacillus plantarum* strain 1625. Front. Microbiol..

